# A qualitative study examining the impact of multidrug-resistant organism (MDRO) carriage on the daily lives of carriers and parents of carriers with experiences of hospital precautionary measures

**DOI:** 10.1186/s13756-022-01141-8

**Published:** 2022-08-13

**Authors:** Ruth Baron, Renske Eilers, Manon R. Haverkate, Sabiena G. Feenstra, Aura Timen

**Affiliations:** 1 National Coordination Centre for Communicable Disease Control (LCI), Centre for Infectious Disease Control (CIb), National Institute for Public Health and the Environment (RIVM), Bilthoven, The Netherlands; 2 Department of Primary and Community Care, Radboud Institute for Health Sciences, Radboud University Medical Center, Nijmegen, The Netherlands

**Keywords:** Multidrug-resistant organisms, Antimicrobial resistance, MDRO carriers, Hospital precautionary measures

## Abstract

**Background:**

Multidrug-resistant organism (MDRO) carriage may have an adverse impact on the quality of life of carriers, in particular those who have experienced hospital precautionary measures. This study aims to gain a deeper understanding of how MDRO carriage has affected the daily lives of carriers with these experiences.

**Methods:**

This was a qualitative study based on 15 semi-structured interviews with MDRO carriers or parents of carriers, which were analysed by thematic analysis.

**Results:**

Three main themes were identified: (1) *Feeling dirty and unworthy* portrays the feelings that MDRO carriers often expressed and how these were related to the language usage describing the MDRO, the perceived avoidance by staff and those in their personal networks, and the effects of the precautionary measures implemented in the hospital. (2) *MDROs are invisible, but impact is visible* covers how the microbe, despite its apparent invisibility, still impacted carriers in their physical and psychological health. MDRO carriage disrupted their lives, by affecting their other unrelated medical conditions at times and by causing varying levels of fear for their own and others’ health. (3) *Carrying the burden on one’s own shoulders* describes the lingering questions, uncertainties and confusion that carriers continued to live with and the perceived burden and responsibility that lay on their own shoulders with respect to carrying and preventing the transmission of the MDRO.

**Conclusions:**

MDRO carriage can negatively influence the quality of people’s lives in various ways. Improved support and sensitivity from health care providers (HCPs) are needed to address feelings of unworthiness among MDRO carriers and the fears that many experience. Clearer information and guidelines are also needed from HCPs to address the many questions and uncertainties that MDRO carriers face outside of the hospital in their daily lives.

## Background

Antimicrobial resistance (AMR) is a major public health issue that is increasingly leading to substantial morbidity and mortality around the world. Estimations for annual deaths resulting from infections due to AMR are over 33,000 in the European Union [1] and over 35,000 in the United States (US) [2]. A recent study estimated that AMR had directly caused 1.27 million deaths and was associated with 4.95 million deaths worldwide in 2019 [3]. Some of the most commonly acquired multidrug-resistant organisms in the Netherlands include *Escherichia coli* (*E.coli*) (colonizes the normal gastrointestinal tract; most frequent cause of bloodstream and urinary tract infections), methicillin-resistant *Staphylococcus aureus* (MRSA) (colonizes the skin and nostrils; can cause bloodstream, skin and bone infections) and *Klebsiella pneumoniae* (*K. pneumoniae*) (colonizes the skin, gastrointestinal and respiratory tract; can lead to urinary tract and lower respiratory tract infections) [4].

Various strategies are required to combat the rising levels of AMR, including developing new antibiotics and vaccines, mitigating inappropriate antibiotic prescriptions by physicians as well as the overuse and misuse of antibiotics by patients, reducing the usage of antibiotics in farming, preventing antibiotic residues from entering the environment, improving the surveillance of AMR, and preventing further transmission by those already carrying resistant microbes to others [5, 6]. The prevalence of AMR is relatively low in the Netherlands. It has been estimated that less than 1% of the population carry the common resistant bacteria MRSA, for instance [7]. Several factors have contributed to a low prevalence of AMR in the Netherlands. The conservative prescription of antibiotics by health care providers (HCPs) and programs such as the One Health approach initiated by the Ministry of Health in 2015 have contributed to curbing the rise of AMR. The defined daily dose (DDD) antibiotic consumption of 8.7 per 1000 persons in the Netherlands is the lowest in Europe [8]. Since 1988, the Search and Destroy Policy has also been implemented in hospitals and nursing homes in the Netherlands. Patients are screened for an increased risk of carrying multidrug-resistant organisms (MDROs) by completing a questionnaire. If identified as being at risk, they are isolated, microbiological cultures are taken, and if necessary, carriers are treated. Although this Search and Destroy Policy may be an effective way of saving medical costs and preventing further morbidity and mortality due to AMR [9], there has been less focus on the consequences of what it means to be a MDRO carrier. MDRO carriers can experience feelings of anxiety, stigma, social isolation and a need for more information [10–12]. Hospital experiences, including precautionary measures and staff behaviours may contribute to a negative impact on the daily lives of carriers [13, 14].

In order to provide MDRO carriers with better support, it is important to gain further understanding of their experiences with being diagnosed as carrying a resistant microbe and how this has influenced their lives. The aim of this interview study is to explore the impact that MDRO carriage has on the daily lives of people who have attended hospital for various medical conditions and have experienced hospital precautionary measures.

## Methods

### Study design

This was a qualitative study making use of semi-structured interviews; almost all of the interviews were carried out with participants face-to-face, one was conducted by telephone. The interviews were conducted between July 2017 and January 2018.

### Study population and recruitment

The study population consisted of participants with the following inclusion criteria: they or their child were either current or recent MDRO carriers (within the previous 3 months), they spoke Dutch and were cognitively capable of participating in the study. Exclusion criteria were working in health care institutions and living in care facilities. Children who carried MDROs in this study were represented by a parent. Due to the difficulty in recruiting sufficient participants, the recruitment involved some convenience sampling. The majority of participants were recruited by contacting microbiologists or infectious disease specialists working in five hospitals in different regions of the Netherlands, most of which have specialized MRSA units. Some HCPs were also contacted as they were part of the network of the department of National Coordination Centre for Communicable Disease Control (LCI) where the researchers work. These HCPs were asked to assist in the recruitment of current or recent MDRO carriers who had attended those hospitals. The HCPs were told about the purpose of the study and that carriers would be asked about their experiences with being carriers and of the care they had received in the hospital. Hospitals either sent the invitation letter (pre-prepared by the research team) to all current and recent carriers, or left the invitation in easily accessible public areas for interested patients to take with them. The invitation letter contained examples of the types of questions that they would be asked, such as ‘How do you feel about being a carrier?’ and ‘What are your views on the information you received about your MDRO carriage?’. Four participants (including the three parents) were recruited through the personal network of the co-authors and by snowball sampling. The authors conducting the interviews and the analyses, however, did not know these participants personally. MDRO carriers (and parents of MDRO carriers) who were interested in participating contacted the research team and an interview place and time were arranged. The participants were gifted a €10,00 voucher afterwards as a token of thanks for their time and effort.

### Interviews and analyses

The interview guide was based on concepts derived from the common sense model of self-regulation (CSM) and the health belief model (HBM) [15, 16]. The CSM is a widely used theoretical framework to explore the cognitive and behavioural processes and perceptions that patients develop when dealing with their disease. The HBM is a widely used theoretical framework to explain and predict health behaviour. In this latter model, the focus is on constructs such as weighing the risks and benefits of the behaviour and self-perceived efficacy. The interview questions were generally open-ended, leaving room for the participants to discuss related topics that were of personal importance to them. Before the start of each interview, the participant was asked to complete a short questionnaire with personal details, including gender, age, main occupation, and how long they had been aware of carrying the MDRO.

The interviews were conducted by one researcher (RE) in the homes of the participants and once by telephone and lasted on average 57 min (range 35 to 87 min). Before the start of the interview, permission was asked from the participants for the interview to be recorded and the data to be published. After RE provided information regarding the process and contents of the interview, participants were asked to sign a form giving their informed consent to participate in the study. Participants were then asked about their personal experiences upon learning they were carrying the MDRO, how they viewed their hospital experiences, the care and advice provided by their HCPs and if and how the MDRO had impacted their current lives. Additional family members, such as the spouse, were also present at times and partook in the conversation. The information provided by these additional members which contributed to understanding the experiences of being a MDRO carrier, was also included in the analyses. The recordings were then transcribed verbatim. A thematic analysis was carried out by RB and RE to analyse the transcripts based on the method described by Clarke & Braun (2013) [17] and Maguire & Delahunt (2017) [18]. This method consists of the following steps: (1) first getting to know the data thoroughly, (2) assigning codes to the data, (3) clustering codes into themes, (4) reviewing these themes, (5) defining and naming these themes and (6) writing out the findings. Steps 3 and 4 included the formation of sub-themes and main themes. Thematic analysis is a flexible method allowing the usage of concepts based on earlier theories and models, as well as new concepts that arise from the data itself. Although theoretical models were used to assist in the development of appropriate interview questions, we did not use these models as a framework by which to analyse the data (e.g. to test the models or to explain behaviours). Instead we took a more inductive data-driven approach, where we allowed the data provided by the participants to help us to identify main themes and subthemes that best represented their experiences. The main themes and sub-themes were discussed between RB and RE to determine whether their interpretations were aligned and to reach agreement on the essential findings. The software MAXQDA 2020 was used to assist in the coding and analysis of the data. Relevant citations were selected and translated into English by RB and are presented in this paper to illustrate each theme. In writing the results section, a balance was sought between providing sufficient evidence for the findings in the data by reporting and discussing individual quotes and keeping the findings as compact as possible by summarizing and paraphrasing quotes of multiple MDRO carriers who had expressed similar sentiments.

## Results

Fifteen interviews were conducted in total: 12 interviews with MDRO carriers in the age range of 42–75 years (average 61.7) and three interviews with parents of children who were MDRO carriers (see Table 1). One of the interviewed parents had two children carrying MRSA and the other two each had one child carrying MRSA. In three of the interviews, the spouses were also present and actively participated in the conversation. Of the 12 adults carrying MDROs, five were MRSA and three were Livestock-Associated (LA)-MRSA carriers. One of the spouses partaking in the interviews was also a MDRO carrier. Relevant quotes by this spouse that contributed to illustrating themes were presented as well. The other four adults were carriers of one or more of the following resistant bacteria: *Escherichia coli (E. coli)*, *Klebsiella pneumoniae (K. pneumoniae), Acinetobacter baumannii* (*A. baumannii)*, *Serratia marcescens* (*S. marcescens*) and *Proteus mirabilis (P. mirabilis)*. Some of the participants had initially experienced physical effects from the bacteria, such as hard-to-treat skin infections, but after these were treated, the majority of participants did not tend to feel any physical discomfort from the MDROs themselves.

**Table 1:** Background characteristics of the study sample

Number of participants	Adult carriers	12 (+ 3 spouses)
	Parents of carriers	3 (4 children)
Age (of adult carriers)	Mean (range)	61.7 (42–75)
Gender	Male (including 1 father)	4
	Female (including 2 mothers)	11
MDRO	MRSA	5
	LA-MRSA	3
	*E.coli*	1
	*K.pneumonia* & *E.coli*	1
	*A.baumannii & S.marcescens*	1
	*E.coli* & *P.mirabilis*	1
	*Children with MRSA*	3 interviews (4 children)
Comorbidity	Cancer	5
	Cardiovascular disease	2
	Diabetes, cardiovascular disease	1
	Respiratory disease	1
	Muscle disease	1
	Incomplete spinal cord injury	1
	Kidney stones	1
	Cleft lip (children)	2
	None (children)	1 interview (2 siblings)

There were 15 interviews with 12 MDRO carriers (+ 3 spouses) and three parents of MDRO carriers

**Fig. 1:**
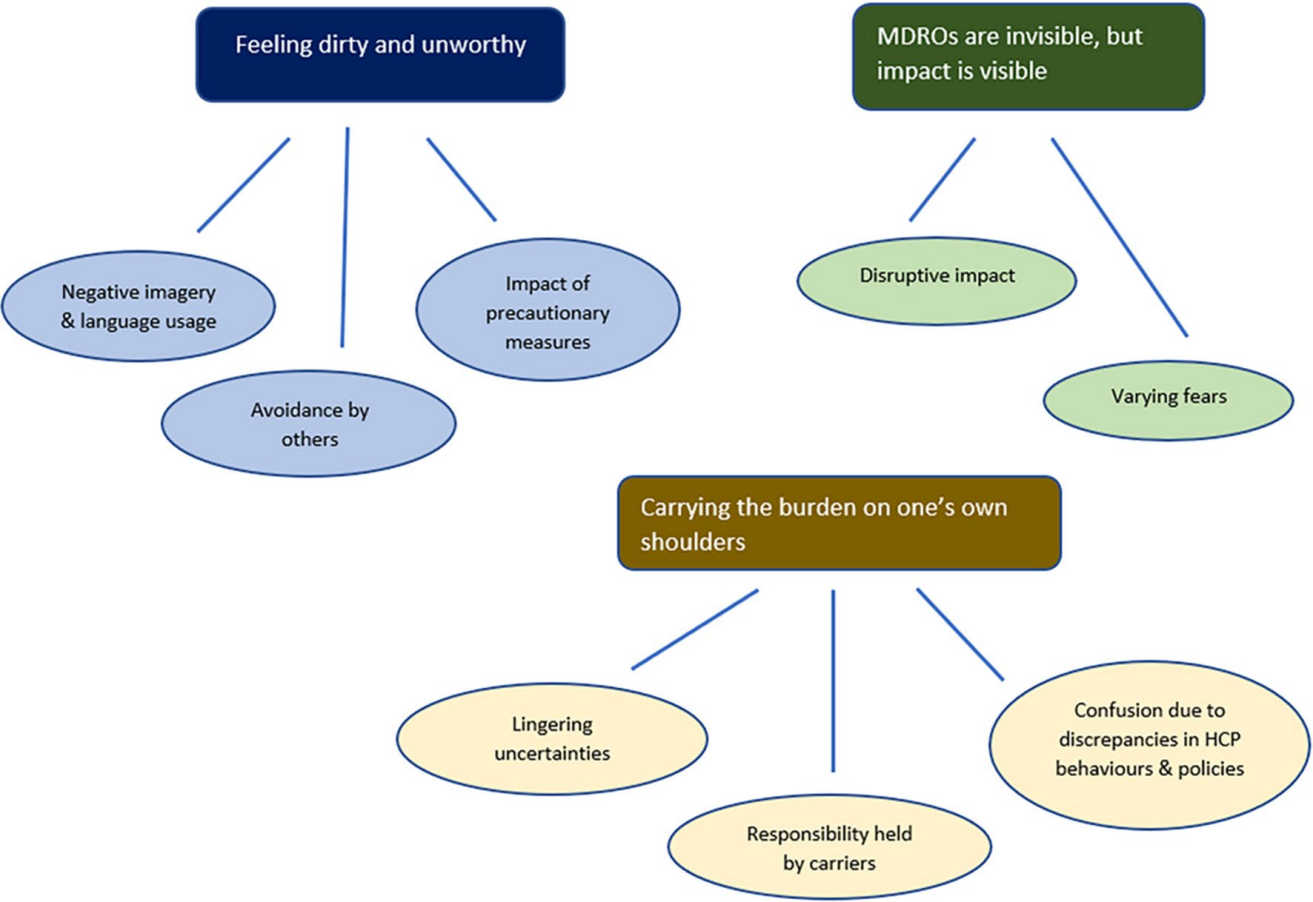
Schematic representation of themes and sub-themes

Three main themes and eight sub-themes were identified that encompass the impact that being a MDRO carrier had on these participants’ lives (see Fig. 1). Theme 1. 'Feeling dirty and unworthy' with the three sub-themes *Negative imagery and language usage*, *Avoidance by others* and *Impact of precautionary measures* covers various ways in which carrying the resistant bacteria made MDRO carriers feel dirty and unworthy. Theme 2. 'MDROs are invisible, but impact is visible' describes how the resistant microbe, despite being invisible, still made an impact on their physical and psychological health, and contains the sub-themes *Disruptive impact* and *Varying fears*. The third theme 3. 'Carrying the burden on one’s own shoulders' covers the questions, uncertainties and confusion the participants continued to live with. This theme also recounts the perception that participants themselves had to carry the burden of MDRO, as well as the responsibility for preventing transmission to others carriage on their own. This theme consists of the sub-themes *Lingering uncertainties*, *Confusion due to discrepancies in HCP behaviours and policies* and *Responsibility held by carriers.* As most participants had spent time in isolation rooms, various aspects of these isolation room experiences reoccur throughout the description of the various themes. The type of MDRO that the participant was carrying is added after each quote.

## Feeling dirty and unworthy

Throughout the interviews, it became apparent that the participants had varying perceptions of the MDRO they were carrying, from having very little psychological impact on some and an intense impact on others. The majority of participants did, however, describe varying feelings of worthlessness, shame, being dirty and looked down upon as if they were lepers and social outcasts. Various topics surfaced during the interviews which were related to these feelings, such as the negative imagery of and language usage when describing the MDRO, the avoidance by hospital staff and the precautionary measures implemented to prevent the spread of the microorganism.

### Negative imagery and language usage

One woman referred to the resistant *E. coli* she was carrying as a monster; she described the feeling of having ‘that animal’ in her as being very stressful. She mentioned that one type of antibiotic she was taking, gave her some control over the bacteria, so that ‘it cannot crawl up inside my body’. One participant referred to the MRSA as ‘that thing in you’. Another participant carrying MRSA described a feeling of having something aversive in her body after seeing the protective clothing worn by a HCP.

*‘The man* (doctor in protective clothing) *came in like that and I just felt dirty. Really dirty. I thought ‘what do I have there in my body?’* Interview 1 (MRSA)

Participants referred to the bacteria as resistant bugs or beasts (translated literally from a Dutch term that can refer to less ferocious animals of different sizes, as well as to beasts) and indicated that HCPs did as well. Although many participants did not mention being bothered by the language used, a few did not always appreciate the way the MDRO was described by some HCPs. The husband of a participant who was also a MRSA carrier himself mentioned that after he had been operated for another medical issue in hospital ‘a doctor walked by me saying ‘so many bugs, so many bugs’. He said it, you know, I dare to swear he did.’ Doctors and patients when referring to being MDRO-free tended to use a Dutch term meaning ‘clean’. Feeling dirty appeared to be compounded by the language usage of being clean when referring to testing negative for the MDRO.

*‘I need to be clean three times, then they will shake your hand and whatever else.’* Interview 1 (MRSA)

### Avoidance by others

Related to feelings of being unclean, participants often expressed the avoidance by both staff in hospitals and others in their social networks. To prevent the transmission of the MDRO to others, participants had to be treated in special isolation rooms. Several participants had experienced extreme loneliness during their stay in the isolation rooms in the hospital, feeling the staff were distancing themselves from them, reinforcing their feelings of being ‘unclean’. One participant mentioned that the staff would call out to her through a round opening in the door instead of coming in to talk to her and how this made her feel.

*‘I was really treated as a leper, and I thought, I can’t help it that I have this and I am lying here just being sick.’* Interview 6 (LA-MRSA)

When she mentioned this issue of feeling avoided to the staff, the situation did improve, however. As several participants mentioned, this avoidance by staff was also likely due to them having to change into protective clothing every time they entered the isolation room, which was time-consuming. Another participant reported having the feeling that the staff would avoid eye contact with her, for fear she would ask them questions. She described the staff as typically entering, putting her food down, then turning around and leaving immediately. She felt she was not asking too much from them.

*‘No one needs to come in to hug me or hold me. No, I would say, just let the person feel like they are a human being.’* Interview 7 (MRSA)

The social impact of carrying MDRO varied among participants, with many reporting worries of infecting others and worries by families and friends of being infected by them. This led to hesitance in going to certain locations and in contacting certain people whom they thought may be vulnerable. Some experienced their friends wanting to keep their distance from them.

*‘I had the feeling when I told people, they did not want to visit. They were afraid to come near me. I felt like an outcast….Another friend of mine, who lives in [location], she invited me to her birthday party. I said we were still dealing with the MRSA. I didn’t need to say anything more, she immediately said ‘then it’s best you don’t come.’’* Interview 7 (MRSA)

Several participants mentioned they didn’t tell anyone about their MDRO carriage anymore to avoid negative reactions.

*‘All of our acquaintances, we don’t tell them. We keep it to ourselves, that MRSA…..even though we are very transparent about other things. But we don’t tell them this. People are shocked, ‘I better not talk to him, otherwise I may get it too.’’.* Interview 1 (Husband: also MRSA carrier)

On the other hand, there were a few participants who had told their family and friends, but had not perceived any negative reactions. These participants also tended to feel less shame or dirty about their MDRO carriage. One participant, for example who worked as a pig farmer just considered his LA-MRSA as a part of the package of his job, and did not consider it to limit him socially.

‘*No, I would not say, ‘my neighbour has a cold, I cannot go and see her now’, no, we don’t see it as something that bad.’* Interview 2 (LA-MRSA)

### Impact of precautionary measures

The isolation room and precautionary measures implemented in the hospital contributed to feelings of being treated as less worthy than other patients. The husband of a participant who was also carrying MRSA mentioned that surgeries (including his own) for MDRO carriers were always scheduled for last.

*‘Surgeries are always at the end of the afternoon. They all come in with white coats and face masks. They don’t like to come in, that is very clear.’* Interview 1 (Husband: also MRSA carrier)

One woman who had stayed in an isolation room, described not being permitted to shower until everyone else had showered in the mornings. Similarly, she could only choose what she wanted for her evening meal after everyone else had made their choice, leaving very few dinner options, and she described the food that she was served as being cold sometimes. These measures reinforced her feelings of unworthiness.

Another woman who had been in an isolation room mentioned she had been lucky to have a glass door, so she could wave and pass messages on notes to the staff. Her husband had also been there to keep her company, and the nurses did come in to talk to her now and again, which she appreciated. However, she mentioned that when she was initially brought to the isolation room, she was told ‘Your door will be locked, as you may not leave the room.’ This experience of being locked in a room made her feel like she was in a prison; she was currently worrying about having to go back behind those locked doors for her upcoming surgery.

Although the majority of participants who had stayed in isolation rooms had expressed discontent with their experiences there, one woman mentioned she had enjoyed being in isolation.

‘*I did not mind, I loved it. I had visitors. When you are there on your own, they (staff) are not difficult. I enjoy watching tv, I didn’t mind. I am someone who enjoys company, but you need to be lucky with whom you end up. And I was very sick, of course, when I was admitted, you don’t always feel like talking and you can do whatever you like*.’ Interview 11 (resistant *E.coli*)

## MDROs are invisible, but impact is visible

The participants sometimes referred to the bacteria as being invisible and elusive, but still clearly having a psychological and physical impact on them.

*‘They say you have it. You don’t feel it, where is it? I don’t know where it is. I really don’t.*’ Interview 5 (MRSA)

One participant mentioned it was better to have a broken arm, as people can clearly see that something is wrong. She compared it to having psychological issues:

*‘When something is in your head, no one sees that. They think everything is going well. I think that is how it is with MRSA as well.’* Interview 1 (MRSA)

### Disruptive impact

Although the microbes generally did not cause physical discomfort and appeared to be invisible, they sometimes had a disruptive impact on other areas of their lives, such as on the comorbidities they were dealing with. One woman described how she had been waiting for a long time for a hip replacement. Her MRSA carriage had led to her appointment being postponed, to prevent any chances of developing an untreatable infection. She explained some of the positive effects the hip surgery would have on her general health and wellbeing.

*‘Look, if they would help me with my hips, I could maybe walk better. Maybe I could cycle again. Then I might lose some weight again. Then I will feel better, because of all this. You become sick, only because of a bacterium.’* Interview 7 (MRSA)

Similarly, participants with children who carried MRSA did not report any discomfort that was directly related to the bacteria. The disruptive impact on their lives was visible, however, through all the rules and regulations surrounding the MRSA carriage. One parent mentioned having to call for the results themselves each time their child was tested. All the parents reported having to go through great lengths to have the MRSA label removed from their children’s medical records, even after they had been tested and diagnosed as being free of MRSA. They were all eager to have the label removed, because of all the precautionary measures that accompanied MRSA carriage. One participant recounted the experience of their older son (who was not a MRSA carrier) being admitted to hospital for a medical issue, and then on arrival unexpectedly being put into an isolation room. This had caused them all a bit of stress. Another mother mentioned that her children had only been tested because a close family member had tested positive for MRSA, but the ensuing medical burden they experienced made her regret the decision to have her children tested.

*‘I was like, ok, that’s fine, but if I had known then what I know now, then I would have preferred them not being tested. Then we wouldn’t have known, and then there wouldn’t have been any consequences. We went along with it without realizing what it was all going to mean.’* Interview 15 (parent of children with MRSA)

### Varying fears

Irrespective of whether the MDRO had led to any physical problems, the resistant microbe still managed to evoke fear in many participants. Participants did vary in the extent of fear they felt, with some being consumed by intense fears and others experiencing no fear at all.

*‘Look, the bacterium itself, I don’t feel it at all, but I want to be rid of the fear. You cannot really explain this to anyone.’* Interview 11 (resistant *E.coli*)

The intense fears were either predominantly about spreading the bacteria to others who were weak or sick, or about the possible effects of the resistant bacteria on their own health. Many participants pre-emptively avoided certain locations or people whom they thought may be susceptible. One woman reported that the hospital had told her she needed to be separated from others in the pulmonary department, otherwise many people would get sick or die. This woman now had excessive fears about infecting her children and other people if she went out somewhere. She made sure her children stayed upstairs in their rooms if they were sick, so she wouldn’t infect them. She would not let anyone use her bathroom and would also not visit a sick friend in hospital. She was afraid to go to the pharmacy in case she infected people there.

*‘I think it is awful, I make sure medicines are delivered to my home, so that I don’t infect sick people there. What if they die because of me? I don’t want that on my conscience.’* Interview 5 (MRSA)

Other participants worried about the effects of the MDRO on their own health, at times fearing it would lead to their own death. One woman who had attended the hospital for breast cancer felt like she had less control and thus a greater fear of the resistant *E.coli* she was carrying than the breast cancer.

*‘I can’t do anything about the bacteria, I can’t say, I will take that cure or that radiation and then it will go away. So I am more afraid of that than of my breast cancer, even though it* (breast cancer) *could come back next year or in five years.’* Interview 11 (resistant *E.coli*)

In contrast, a few participants did not express having much fear of the MDRO. The serious comorbidities they were battling, appeared to have taken much more of a toll on their lives. One woman was asked how serious she considered her LA-MRSA to be.

*‘Oh, I don’t mind it at all, it makes no difference to me. No, it does not keep me awake at night. I think they will always be able to help me, so I have quite a positive attitude in that way.’* Interview 6 (LA-MRSA)

When asked if she was worried about passing the MDRO on to others, she responded that she never had time for such worries, as she was focused on her own survival. When a male participant who had been treated for cancer was asked if he was interested in knowing whether he was still carrying the MDRO, he responded ‘I don’t know. Is it important that I know?’. The MDRO was just a side-issue of minor importance to him. Another male participant with cancer mentioned he had never even thought about the MDRO anymore after coming home from the hospital, and only thought about it again when he was invited for the interview. The parents of MRSA carriers also did not express fear of MDRO carriage for their own children’s health and for others that were healthy. When one parent was asked if they were worried about becoming MRSA carriers themselves, he replied ‘not at all’. As they were healthy, it was not an issue for them.

*‘Maybe half of the Netherlands is a carrier and no one knows it. It is questionable how serious it really is.’* Interview 3 (parent of child with MRSA)

## Carrying the burden on one’s own shoulders

Besides the burden felt by many participants for the reasons described before, there was also a sense at times that the responsibilities associated with carrying the MDRO were placed on their own shoulders. They also felt they had been left alone to deal with the consequences of MDRO carriage.

*‘You want to hear from someone. But what can they tell you? You have it (the MRSA) and there is nothing that can be done about it. They can’t give you a pill. They can’t give you advice, ‘You need to do this. You should eat this’. I mean, there is nothing. You are left to your fate. That’s it, because there is nothing they can do about it.’* Interview 1 (MRSA)

### Lingering uncertainties

Participants generally confirmed that they had received basic verbal and written information from the hospital regarding MDRO carriage, but many wished they had received more information.

There were questions shared by most participants that still appeared to linger in their minds and continued to cause some frustration. One of the most common questions they struggled with was where they had acquired the resistant microbe.

*‘Where did I pick this up? Did I already have this? Or did this happen in the hospital? How long have I had this in my body? You never find out, I find that so strange.’* Interview 1 (MRSA)

Another participant who was constantly wondering where she had acquired the resistant bacteria was told by the hospital that she hadn’t been near anyone in the hospital from whom she could have gotten it, that it was likely due to her weak immune system, and she could have acquired it from anywhere. She still found that hard to believe.

*‘I say (to the hospital), but you mention farms, I have not been near farms, I have not been in hospitals in other countries, I don’t know anyone in another country that I have had any close contact with.’* Interview 7 (MRSA)

She concluded she must have gotten it from her dog. Another participant concluded he must have gotten it from his cat. Not knowing where they had acquired the resistant microbe caused frustration in many participants. As one woman explained, if you only knew where you had picked it up, then you would also know what to avoid in the future. Another frustration many participants shared was not knowing how long they would be carrying the MDRO; its presence seemed to be unpredictable. The woman waiting for the hip replacement that continued to be postponed, mentioned feeling depressed due to this unpredictability. Another woman described this uncertainty as leading to confusion.

*‘I constantly do that test, I have it* (the MDRO) *and then I don’t have it. I don’t understand why that is……you think you don’t have it and then you’re in the hospital and they test again and you have it again. I find that confusing.’* Interview 5 (MRSA)

Another very common question expressed by the majority of participants was which people and places they should be avoiding to prevent the spread of the MDRO. They wondered how infectious they really were.

*‘I think, yes, I don’t know with whom I should be careful. I would like to know more about that, then I know, oh yes, be careful if I go there.’* Interview 7 (MRSA)

One of the parents whose child was a MRSA carrier said that although she knew a lot about the bacteria, the type of information she really lacked was more of a practical nature, such as whether it was safe for others if they went shopping. One woman described how people often worried about visiting her, because of the MRSA. She said people in general didn’t understand how infectious she was to them and whether it was safe to come near her. She added that she herself was not really sure either. She wished there was some information available about what to tell others.

*‘It would be nice, if you are a patient, that there is such a brochure, a brochure about what to do about people who visit me and who say ‘I don’t dare to come, because you have MRSA’. How do I explain this* (the MRSA)*? That is just difficult.‘* Interview 6 (LA-MRSA)

### Confusion due to discrepancies in HCP behaviours and policies

Besides the lingering questions participants were living with, the participants also reported feeling confused by discrepancies in the behaviours of hospital staff and policies with regard to precautionary measures. Many participants were already wondering how infectious they really were for others; the discrepancies they observed among health care providers and other hospital staff with respect to applying precautionary measures, such as protective clothing, appeared to reinforce their confusion.

*‘What I found odd in (name of hospital), there was a cleaner who came in completely covered in protective clothing to clean, ten minutes later the surgeon came in and shook my hand. I said ‘wait a minute, this doesn’t make sense. I just saw the cleaner completely covered and I receive a handshake from you.’ ‘Ah, I will wash my hands in a moment‘ he told me’.* Interview 6 (LA-MRSA)

Hospitals also varied in the policies they had regarding matters such as the required number of times patients should have a negative test, before having the label of MDRO carriage removed from their dossiers. One participant mentioned that in one hospital she had already been declared free of resistant *K. pneumoniae*, but in another hospital they continued to apply protective measures and to treat her as a carrier of resistant *K. pneumoniae*. The hospital had been unwilling to test her and she felt the staff generally did not seem to have much knowledge about the bacteria.

### Responsibility held by carriers, or their parents

There was a general feeling among many participants that the responsibility for the burden of carrying and preventing the transmission of the MDRO lay on their own shoulders. Several participants mentioned the confusing contrast between all the restrictions in the hospital and being told they could go home and live relatively normal lives. In line with their lingering questions about who they should avoid, many participants wondered why all these precautionary measures were only of importance to patients in the hospital and there was no apparent concern about them infecting people outside of the hospital with potentially weaker immune systems. One woman carrying the resistant *K. pneumoniae* bacteria was worried about how contagious she was to her grandchildren and her pregnant daughter-in-law. She mentioned that the information she had received from the hospital was not clear.

*‘I said* (to the staff)*, you are all completely protecting yourselves, so that other people will not be infected, so that I will not infect other people. But what about my own living environment? And yes, they didn’t know the answer to that.’* Interview 13 ( resistant *K. pneumoniae* & *E.coli*)

Another woman found it strange that she was told not to worry about infecting her husband with MRSA.

*‘They* (HCPs) *were both fully covered in protective clothing. And then I said, ‘But how does this work? My husband is also here, open and bare’. ‘Oh that is no problem. It’s only a problem for us’, they said.’* Interview 1 (MRSA)

When her husband later turned out to be carrying MRSA as well, she had persistent feelings of guilt that she had possibly infected him: ‘I thought it was awful. I wonder, did I do that? Is that my fault?’. She also wondered whether the information she had received regarding not having to worry about infecting her husband, who also had other medical conditions, could be trusted.

Although the perceived concern was mainly for patients in the hospital, some participants felt at times that they also had to warn the staff themselves about their MDRO carriage. A mother reported feeling the hospital staff were sometimes a bit slack with regard to protecting other children in the hospital, coming in for example to take away the plate of food, without changing into protective gear. One woman described her partner having to warn other patients in a waiting room of the hospital, including one patient with cancer, not to sit next to her, even though the staff at the counter were aware she was carrying MRSA. A father mentioned having to warn the health care staff themselves that their child was carrying MRSA and that he needed to be isolated.

(To the hospital staff) *‘Yes, but he is MRSA-positive, so he really must go to the infection room.’ ‘Oh yes, it is good you mention that.’ This happened very often, and yes, this really surprised me, it is not all as watertight as one would hope it to be.’* Interview 3 (parent of child with MRSA)

## Discussion

### Main findings

This study aimed to gain further understanding of the impact that MDRO carriage and the experiences of hospital precautionary measures have on the daily lives of carriers. Many participants expressed feelings of shame, being dirty and of being treated like lepers and outcasts. The language used by both the participants and HCPs to refer to the resistant bacteria may have made these bacteria appear bigger and dirtier than other bacteria. The avoidance of staff and others in the personal networks of the participants, as well as the precautionary measures (including the hospital isolation rooms and protective clothing) added to these feelings of unworthiness. Participants often described the resistant microbe as being invisible, but that its impact was present in other areas of their lives including their physical and psychological health. There were variations in the extent of fear of the MDRO, from no fear at all to intense fears of how the bacteria would affect their own and others’ health. The majority of participants were dealing with uncertainties and lingering questions about the MDRO they were carrying. Questions included where they had acquired the microbe, how long they would be carrying it, how infectious they were and who they should be avoiding. There was much confusion due to observed discrepancies in HCP behaviours and hospital policies regarding precautionary measures. Finally, many felt that the responsibility and the consequences of MDRO carriage were largely placed on their own shoulders, which they had to bear on their own.

Some of these findings confirm earlier findings on the experiences of MDRO carriers. Similar feelings of shame, isolation, being unclean and rejected, having fears of infecting others and a need for more information were reported in a systematic review on the experiences of carriers [12]. A Dutch qualitative study among nurses carrying MRSA also reported them feeling stigmatized and having worries about infecting others [19]. Less attention, as far as we are aware, has been given to the finding in our study that language usage describing these bacteria appeared to be associated with the feelings of being dirty or less worthy. Resistant bacteria are often called ‘superbugs’ in scientific articles and in the media [20–22]. Its function is likely to evoke the urgency of AMR and to mobilize people into addressing the issue [23]. While usage of these terms may be beneficial for those purposes, sensitivity to language usage (e.g. ensuring carriers do not envision their bodies as being infested with large bugs) is likely needed toward those already carrying resistant microorganisms.

Other factors reinforcing feelings of unworthiness and of causing aversion in other people were the avoidance by staff and those in their social networks. Studies have shown that patients in isolation actually do tend to have fewer and shorter visits by HCPs than those in standard hospital rooms [24, 25], indicating these feelings of avoidance may not be subjective. These negative experiences after being diagnosed with carrying an MDRO may lead to a changed body image [13]. Carriers may now need to navigate their lives with a new self-identity, that of a person carrying a contagious, potentially harmful microbe, of which they don’t know its source nor the length of time it will remain in their body. Special care should therefore be taken to ensure that patients carrying MDROs are given the attention they need. Those that may already be enduring other serious comorbidities may be in need of even more care and support. Another finding expressed by many participants in our study, that has not been given much attention before is the odd discrepancy with regard to all the precautionary measures being implemented in the hospital to protect other vulnerable patients, and the lack of precautionary measures recommended to them outside of the hospital. They were told they could lives their lives normally when they got home without the worry of infecting others. The participants in this study tended to be older and therefore more likely to have spouses and other vulnerable social contacts with weaker immune systems, but they appear not to have been given adequate advice on how to handle this situation after exiting the hospital doors.

### Implications for health care and further research

Although most participants remembered receiving some information from their HCPs about MDRO carriage at some point, this information may be insufficient. People’s questions on where and how they acquired the MDRO, how long they would have to live with it, and how they can minimize the risk of recolonization in the future should be adequately addressed. As studies have shown that MDROs, such as MRSA, do spread easily amongst household members [26, 27], follow-up care and guidance is needed to reassure and advise those living with fears about transmitting the resistant microbe to other vulnerable people in their personal environment. Rump et al,. (2020) [28] advocate collective responsibility towards carriers through measures, including assigning a case manager to patients while under medical care, as well as when leaving the hospital, to ensure continued care and support. HCPs should align in their treatment of MDRO carriers, according to existing protocols [29], particularly with regard to the protective gear they wear, to reduce the confusion caused by discrepancies. All staff should ideally be aware of the correct procedures regarding the treatment of MDRO carriers, so that these patients do not feel the responsibility for preventing the spread of the MDRO to others in the hospital to lie on their own shoulders. It may be beneficial to include more training to HCPs about how to handle and support those patients. Guidelines such as The WHO Health Workers’ Education and Training on Microbial Resistance: curricula guide (2019) [30], for example, focus on the knowledge and skills needed in various HCPs for the prevention of infectious diseases and AMR, but it may also be beneficial to provide training on how to care for, support and advise those already carrying resistant microbes.

Further research on the views of HCPs treating MDRO carriers would be beneficial to gain more insight on how MDRO carriers could be supported further in coping with their uncertainties and feelings of unworthiness, confusion, worries and fears. It would also be beneficial to conduct a similar study among a population of MDRO carriers, who are not dealing with such serious comorbidities, to determine the extent that these issues affect all carriers irrespective of their health. For more targeted support, research could focus on examining the demographic and medical characteristics of those more prone to negative experiences.

The world is currently in the midst of the COVID-19 pandemic, the effects of which on AMR are still to emerge in the following years. There are signs that COVID-19 may lead to increased AMR in many countries, due to the disruption of health care programs targeted towards eliminating AMR, disruption of antibacterial treatments for patients and increased antibiotic consumption in COVID-19 patients [31–33]. Reasons for increased antibiotic consumption, despite COVID-19 being a viral infection, include misdiagnosis as a bacterial infection and the development of co-morbidities in COVID-19 patients, requiring antibiotics. It would be of interest to examine whether and how the appearance of COVID-19 with all its preventive measures impacting the general population may have influenced the prevalence of MDROs in the population, as well as current perceptions of MDRO carriage and precautionary measures for MDROs by people in general, including MDRO carriers themselves. Table 2 provides a summary of health care and research recommendations.

**Table 2:** Summary of health care and research recommendations

Feeling dirty and unworthy	
	Be mindful of language usage when describing MDRO to carriers
	Ensure that patients do not feel like lepers and outcasts by avoiding them, or serving them last
	Mitigate loneliness by ensuring regular contacts between staff and patients
	Ensure that patients do not feel imprisoned in isolation rooms
	Take into account that MDRO carriage is an additional affliction often compounding other serious medical conditions, and that carriers may need all the support they can get
MDROs are invisible, but impact is visible	
	Acknowledge the fears that patients have and provide support and advice
	Minimize the disruptive effects that MDRO status can have on people’s lives, for example by facilitating efforts to remove the status of MDRO carriage from their dossiers
Carrying burden on one’s own shoulder	
	Provide clear information (written and verbal) to carriers on what is currently known and unknown about MDRO carriage, including what to tell their network of family, friends and acquaintances regarding their infectiousness
	Acknowledge the confusion due to discrepancies in behaviours/ policies and align these behaviours and policies where possible
	Improve the general knowledge of staff dealing with MDROs, so that everyone is on the same page
	Provide follow-up care for patients beyond the hospital doors into their further lives
	Consider that negative experiences associated with MDRO carriage may lead to less transparency by MDRO carriers and hesitance in being tested for resistant microbes
Further research	
	Conduct qualitative research with MDRO carriers in the general population representing a younger and healthier population
	Conduct qualitative research with HCPs who treat MDRO carriers to discuss the perspectives expressed by MDRO carriers and possible solutions
	To enable more targeted support, investigate the associations of MDRO characteristics (including demographics, other medical conditions and types of MDRO) with experiences and perceptions, including fear and shame
	Examine the impact of COVID-19 on the prevalence of MDRO carriers as well as on perceptions of precautionary measures and of being a carrier

If antibiotic resistance continues down its current course, the impact will be huge on worldwide mortality rates and will have substantial detrimental effects on health care costs and the general economy of countries [34]. The annual mortality rate is predicted to be about 10 million by 2050 if insufficient action is taken [35]. It is, therefore, paramount that AMR continues to be addressed quickly and comprehensively, including applying the policies that actively identify and treat MDRO carriers. The side-effects of these policies should not be neglected, however. These should be addressed, not only to improve the quality of life for MDRO carriers, but also to encourage the willingness to be tested for MDROs and transparency about carrier status, both of which are important for addressing AMR.

### Limitations and strengths

The majority of the participants had been hospitalized for serious co-morbidities, including cancer and cardiovascular diseases and were somewhat older (mean 61.7 years of age). We cannot verify whether their perceptions of MDRO carriage reflect carriers who are younger, less ill, and have not experienced hospital measures. This study does attempt to give a voice, however, to those who are more vulnerable because of their comorbidities, and who are perhaps in even greater need of supportive health care.

It is unknown whether the convenience sampling that occurred during recruitment resulted in any bias with respect to the characteristics of the participants and the information provided. We believe this was likely to be minimal, considering the diversity of participants we still obtained with regard to age, type of MDRO and type of medical condition. This diversity offered a range of multiple perspectives and experiences. As the participants were informed about receiving the 10€ voucher before agreeing to the interview, we also cannot determine with any certainty whether this played any role in their willingness to participate. In our view, the value of the voucher was too low to have influenced participation, especially in light of the gravity of the topics. The researchers’ background in working for the National Institute for Public Health and the Environment (RIVM), may have influenced the type of information provided by the participants. As this is a well-known institution by most people in the Netherlands with regard to forming policies and conducting research at the national level, their responses may have ranged from being more socially desirable to being especially critical. We believe this influence was unlikely however, as the participants appeared to be speaking openly, honestly and without hesitance about personal and at times intense and emotional experiences.

### Conclusions

An important strategy contributing to the combat of AMR is to prevent the transmission of resistant microbes. The consequences of this strategy for MDRO carriers should not be disregarded, however. MDRO carriage can have an adverse impact on the quality of people’s lives in various ways by causing the following: feelings of unworthiness; fears and worries of varying intensity; lingering unanswered questions; confusion due to discrepancies in HCP behaviours and hospital policies; stress due to precautionary measures and other regulations surrounding MDRO carriage; disruptions to other medical conditions and the perception of carrying the burden of responsibility for the MDRO on their own shoulders. Improved support and sensitivity from HCPs are needed to address feelings of unworthiness among MDRO carriers and their fears for their own health and of transmitting the microbe to vulnerable others. Clearer information and guidelines are needed from HCPs to address the many questions and uncertainties that MDRO carriers face outside of the hospital in their daily lives.

## Data Availability

Due to privacy reasons, participant transcripts cannot be made available. Please contact the authors for any questions regarding the data.

## Abbreviations

MDRO: Multidrug-resistant organism
HCP: Health care provider
AMR: Antimicrobial resistance
MRSA: Methicillin-resistant *Staphylococcus aureus*
LA-MRSA: Livestock-Associated MRSA
*E. coli*: *Escherichia coli*
*K. pneumoniae*: *Klebsiella pneumoniae*
*A. baumannii*: *Acinetobacter baumannii*
*S. marcescens*: *Serratia marcescens*
*P. mirabilis*: *Proteus mirabilis*
DDD: Defined daily dose
CSM: Common sense model of self-regulation
HBM: Health belief model
